# Large-Scale Evaluation of a Rapid Fully Automated Analysis Platform to Detect and Refute Outbreaks Based on MRSA Genome Comparisons

**DOI:** 10.1128/msphere.00283-22

**Published:** 2022-10-26

**Authors:** Kathy E. Raven, Eugene Bragin, Beth Blane, Danielle Leek, Narender Kumar, Paul A. Rhodes, David A. Enoch, Rachel Thaxter, Nicholas M. Brown, Julian Parkhill, Sharon J. Peacock

**Affiliations:** a Department of Medicine, University of Cambridge, Cambridge, United Kingdom; b Next Gen Diagnostics (NGD), Cambridge, United Kingdom; c Wellcome Sanger Institute, Hinxton, Cambridge, United Kingdom; d Clinical Microbiology and Public Health Laboratory, UK Health Security Agency, Cambridge, United Kingdom; e Department of Veterinary Medicine, University of Cambridge, Cambridge, United Kingdom; Escola Paulista de Medicina/Universidade Federal de São Paulo

**Keywords:** MRSA, genome, infection prevention and control, outbreaks, sequencing

## Abstract

Genomic epidemiology of methicillin-resistant Staphylococcus aureus (MRSA) could transform outbreak investigations, but its clinical introduction is hampered by the lack of automated data analysis tools to rapidly and accurately define transmission based on sequence relatedness. We aimed to evaluate a fully automated bioinformatics system for MRSA genome analysis versus a bespoke researcher-led manual informatics pipeline. We analyzed 781 MRSA genomes from 777 consecutive patients identified over a 9-month period in a clinical microbiology laboratory in the United Kingdom. Outputs were bacterial species identification, detection of *mec* genes, assignment to sequence types (STs), identification of pairwise relatedness using a definition of ≤25 single nucleotide polymorphisms (SNPs) apart, and use of genetic relatedness to identify clusters. There was full concordance between the two analysis methods for species identification, detection of *mec* genes, and ST assignment. A total of 3,311 isolate pairs ≤25 SNPs apart were identified by at least one method. These had a median (range) SNP difference between the two methods of 1.2 SNPs (0 to 22 SNPs), with most isolate pairs (87%) varying by ≤2 SNPs. This similarity increased when the research pipeline was modified to use a clonal-complex-specific reference (median 0 SNP difference, 91% varying by ≤2 SNPs). Both pipelines clustered 338 isolates/334 patients into 66 unique clusters based on genetic relatedness. We conclude that the automated transmission detection tool worked at least as well as a researcher-led manual analysis and indicates how such tools could support the rapid use of MRSA genomic epidemiology in infection control practice.

**IMPORTANCE** It has been clearly established that genome sequencing of MRSA improves the accuracy of health care-associated outbreak investigations, including the confirmation and exclusion of outbreaks and identification of patients involved. This could lead to more targeted infection control actions but its use in clinical practice is prevented by several barriers, one of which is the availability of genome analysis tools that do not depend on specialist knowledge to analyze or interpret the results. We evaluated a prototype of a fully automated bioinformatics system for MRSA genome analysis versus a bespoke researcher-led manual informatics pipeline, using genomes from 777 patients over a period of 9 months. The performance was at least equivalent to the researcher-led manual genomic analysis. This indicates the feasibility of automated analysis and represents one more step toward the routine use of pathogen sequencing in infection prevention and control practice.

## INTRODUCTION

Genomic epidemiology is on the cusp of being introduced into routine hospital infection prevention and control (IPC) practice. Methicillin-resistant Staphylococcus aureus (MRSA) is a globally leading cause of death due to antimicrobial resistance (1) and represents an important early adoption case to detect and reduce nosocomial transmission. The combination of MRSA genome and patient movement data provides a more accurate determination of transmission events and outbreaks than standard infection control methods alone (2, 3). Such genomic epidemiology can refute outbreaks and support highly targeted interventions to bring confirmed outbreaks to a rapid close (2, 3). There is also evidence for the effectiveness of prospective routine genomic surveillance, in which all MRSA isolated in the clinical laboratory are sequenced to detect and refute outbreaks at the time of bacterial isolation (4, 5). However, undertaking genomic analyses at sufficient pace to inform the daily decisions made by IPC teams necessitates fully automated interpretation tools.

We previously reported the detection and curtailment of an outbreak in a pilot study of 17 MRSA genomes in which genome analysis was achieved using the Next Gen Diagnostics (NGD) bioinformatics system (6). On completion of the sequencing run (MiniSeq, Illumina), this fully automated system uploads raw fastq folders via an encrypted secure upload to the Amazon UK Web Services cloud, where 60 compute nodes are automatically activated. Layers of quality control, read trimming, assembly, mapping, and relatedness determination to support transmission detection are automatically performed and genome relatedness defined between isolates in that run, and with the entire library of previously processed isolates. These computations take an average of 35 min for a single run, after which results are downloaded to the NGD database for display and use. Analysis includes bacterial species, multilocus sequence type, genome relatedness determinations, the presence of a *mec* gene, and highly accurate (7) antibiotic susceptibility predictions for 31 agents. During the previous pilot study, the automated tool identified an outbreak linked to a ward and an otherwise unsuspected clinic associated with diabetic patient care (6).

An automated analysis tool has the advantage that results are generated without the need for an operator with any bioinformatic training. It would also provide greater standardization of analysis and less interoperator variability compared with research pipelines (8). Here, we extend our evaluation to a 9-month evaluation of MRSA acquisition and outbreak detection in a cohort of 777 patients, which was completed prior to the onset of the COVID-19 pandemic.

## RESULTS

### Overview.

Our aim was to validate the accuracy of a fully automated MRSA genome analysis pipeline. Acknowledging the absence of a “gold standard reference” against which new genome analysis tools can be assessed, we compared its performance against a manual bioinformatics approach (“pipeline”) developed in our research laboratory for bespoke analysis.

A total of 789 isolates were sequenced over 43 sequence runs. Thirty isolates failed the manual pipeline quality control (QC) metrics, 21 of which also failed the automated tool QC metrics. On further inspection, the nine discrepancies (fail/pass) had >10% contamination with another species or high numbers of heterozygous sites (not detected by the automated pipeline QC) (Table S2). Twenty-two of the 30 isolates were resequenced and passed the QC of both pipelines (Table S2) resulting in a total of 781 isolates from 777 patients that were used in further analyses. Most MRSA isolates were from multisite (nose, throat, groin, and/or axilla) screens (*n* = 526), the remainder being from clinical specimens (*n* = 255). Metrics used in the comparison were species identification, detection of *mec* genes, assignment to STs, and genetic relatedness based on pairwise SNPs. Outputs were generated independently for both methods, with blinding of the results of the other method prior to comparison of findings. We defined putative patient clusters based on genomic findings.

10.1128/msphere.00283-22.2 TABLE S2 Details of sequencing metrics for 30 MRSA isolates for which genomes failed manual (*n* = 30) or automated tool QC metrics (*n* = 21/30). Download Table S2, XLSX file, 0.01 MB. Copyright © 2022 Raven et al. 2022 Raven et al. https://creativecommons.org/licenses/by/4.0/ This content is distributed under the terms of the Creative Commons Attribution 4.0 International license.

### Comparison of analysis methods.

The two analysis methods produced fully concordant results for species identification (S. aureus), the detection of a *mec* gene (*mecA* in 776/781 [99.4%] cases, *mecC* in 5 cases), and ST assignment (Table S1) with one exception. One isolate was assigned to ST4671 by the manual pipeline but was termed novel by the automated tool. This was because automated analysis was completed earlier than the research analysis, and the ST for this isolate has not yet been assigned a number in the pubMLST database. A total of 66 STs were identified, with ST22 (*n* = 368; 47.1%), ST45 (*n* = 81; 1.3%) and ST59 (*n* = 61; 0.8%) proving the most common.

10.1128/msphere.00283-22.1 TABLE S1 Details of MRSA isolates used in the study, including genome accession numbers, QC metrics for detection of the *mec* gene, multi-locus sequence type, and notation of isolates involved in outbreaks described in two case studies. Download Table S1, XLSX file, 0.1 MB. Copyright © 2022 Raven et al. 2022 Raven et al. https://creativecommons.org/licenses/by/4.0/ This content is distributed under the terms of the Creative Commons Attribution 4.0 International license.

**FIG 1:**
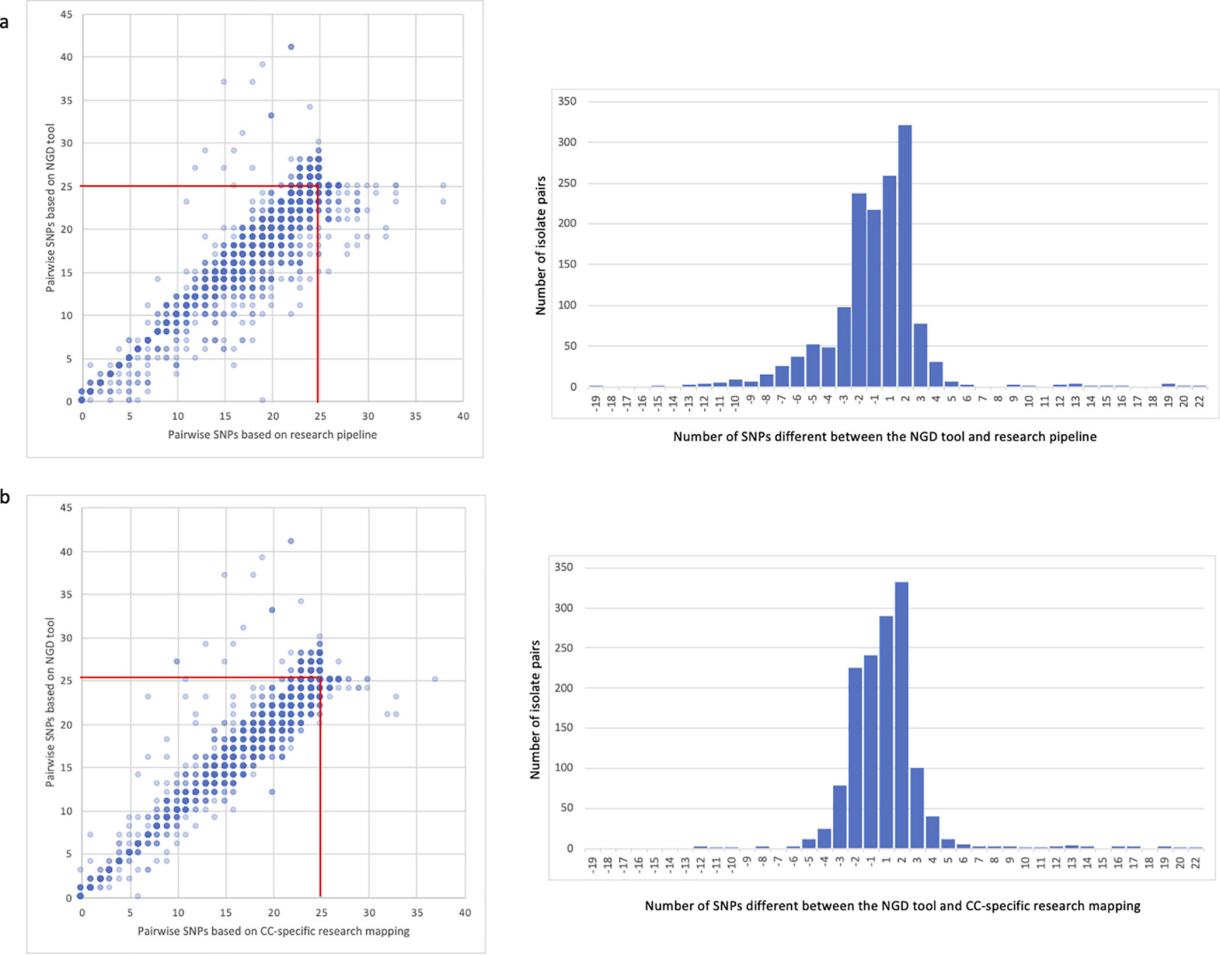
Comparison of genetic relatedness based on the researcher-led manual pipeline and NGD tool for isolate pairs within 25 SNPs of each other based on (a) either the manual pipeline or NGD tool, and (b) either the manual pipeline using CC-specific mapping or the NGD tool for the 83% of isolates belonging to STs with >10 isolates in the collection. Left hand side shows a comparison of all isolate pairs within 25 SNPs. The red box indicates the 25 SNP cutoff used for clustering. Right hand side shows the number of SNPs different between the two analysis methods.

Analysis of genetic relatedness was based on genomes that in a pairwise comparison were ≤25 SNPs apart based on at least one of the pipelines (Figure 1a). This degree of relatedness has been reported as a robust inclusive cutoff for MRSA outbreaks in our setting previously (9). We identified 3,311 isolate pairs that were ≤25 SNPs apart based on at least one of the analysis methods (representing 1% of the 304,590 unique pairs). Of these, 90% (2,981 pairs) were defined as “related” (within 25 SNPs) by both methods, 7% (229 pairs) by the manual pipeline alone, and 3% (101 pairs) by the automated pipeline alone. There was a median (range) of 1.2 SNPs (0 to 22 SNPs) different between the output of the two pipelines and SNP results were within 0 to 2 SNPs for 87% of these 3,311pairs. Of the 330 discrepancies, over half (187, 56%) were due to “minor” SNP distance differences (≤2 SNPs) that were around the 25 SNP cutoff boundary.

### Identifying patient clusters.

We combined the genomic relatedness data for the 3,311 case pairs with their epidemiological data (hospital admission and ward movements [if an inpatient at CUH], general practitioner, and residential postal code). An epidemiological link was made between each pair with genetically related isolates when either a case-pair was admitted to the same ward with overlapping dates of admission or within 7 days of each other in the past 6 months, or if cases shared a postcode or GP practice. Both the manual and automated method identified the same 66 clusters involving 338 isolates/334 patients. Moreover, both methods defined the same cluster sample composition with respect to samples present in each cluster.

While no additional clusters were identified by the manual analysis alone a further four genetic clusters involving nine patients were identified by the automated tool alone. The SNP distance between the isolate pairs was close to the cutoff (range 21 to 25 SNPs). The timing between the positive samples was often protracted. None of the four clusters had relevant epidemiological links. Two further clusters were not clear-cut in their interpretation and were considered “indeterminate.” One cluster of 25 isolates/patients identified by the manual pipeline contained a further 2 isolates identified by the automated tool alone. These two isolates only just reached the cutoff threshold (23 to 25 SNPs) and were 26 to 29 SNPs from the cluster using the research pipeline. The timing between the positive samples in the cluster spanned more than 250 days, and the patients shared a community epidemiological link. The second indeterminate cluster involved nine isolates/patients called by the manual pipeline, which was split into two clusters separated by 29 SNPs (above the cutoff) and a singleton by the automated tool. This cluster spanned more than 100 days and there were no epidemiological links.

### Analysis of differences in SNP calling between the two analysis methods.

To explore whether SNP calling discrepancies between the two pipelines were a feature of mapping to a distant reference, we repeated the manual pipeline analysis using mapping to clonal complex (CC)-specific references for any ST with more than 10 isolates (*n* = 655, 83%). A total of 3,289 pairs were ≤25 SNPs apart based on CC-specific mapping or the automated tool, with a median (range) of 0 SNPs (0 to 22 SNPs) different between the two methods (Figure 1b). SNP results were within 0 to 2 SNPs for 91% pairs (2981/3289), indicating a modest improvement in agreement.

Isolate pairs >10 SNPs different between the automated pipeline and CC-specific manual pipeline (*n* = 21) were investigated further. There were 66 SNP locations identified by the automated tool but not mapped by the manual pipeline, which called an “N” (uncertain/absent base) at variable positions due to no reads mapping (these occurred over an ~450bp region with a transposase fragment (*n* = 19 SNP locations) and an ~8.5 kb region, including an LPXTG surface protein and a bone sialoprotein binding protein (*n* = 3 SNP locations)); short segments mapping in regions of the genome without any other mapping (*n* = 30 SNP locations); and low (2×) depth of coverage (*n* = 2 SNP locations). The basis for the other mismatches was unclear, although some appeared to have a mixture of base calls present in the bam file (*n* = 3 SNP locations).

There were 38 SNP locations identified by the manual pipeline but not by the automated tool. Seven mismatches appeared to be genuine SNP locations that were not detected by the automated tool. This was due to an error in the code such that when both isolates had a different SNP compared to the reference at the same location, or multiple alternative alleles were identified for a position, the SNP was not identified. This has been rectified in an updated version. Manual analysis of genome data identified several explanations for the remainder, including likely misalignments near to indel sites (*n* = 24), and SNP locations relating to a mix of bases called in the bam file that were identified as a single base by the manual pipeline during variant calling (*n* = 6).

### Two case studies.

The genomic data were generated under research ethics and were not made routinely available to the IPC team, but isolate relatedness data were released on request. Here, we describe two case studies in which IPC decisions were influenced by these data.

**(i) Preventing unnecessary infection control escalation.** Five patients in a neonatal intensive care unit were found to be MRSA positive over 4 months, four of which were within a 5-week period and were considered putatively linked based on time and place. Plans were developed for an urgent outbreak response, including an incident management meeting involving senior staff, staff MRSA screening, and additional hand hygiene and cleaning audits. However, sequencing and the rapid analysis enabled by the automated tool showed that the five isolates were unrelated (>495 SNPs different from each other). The planned outbreak response, including any related costs, resources and disruption, was deescalated.

**(ii) Supporting targeted infection control interventions.** The IPC team investigated 5 MRSA-positive patients flagged over a 3-week period on a single ward. Four cases were in a four-bed bay, and the fifth case was elsewhere on the ward. The near real-time availability of strain relatedness information allowed IPC to recognize that all patients in the 4-bed bay carried the same MRSA strain, while the isolate from the 5th case was unrelated. This supported a targeted intervention in which the single bay was closed and cleaned; exclusion of case 5 prevented full ward closure and cleaning.

## DISCUSSION

Here, we describe the findings of an evaluation of an automated bioinformatics system that undertakes real-time analysis of MRSA genome data to determine pathogen relatedness to provide information to confirm or exclude an outbreak. Outputs were compared with a research-led manual bespoke analysis approach that was time-consuming and required an experienced bioinformatician. The automated tool was 100% accurate in assigning species, ST and *mec* genes compared to the research pipeline. There was a high degree of concordance between SNP calls, and between the designation of isolate pairs based on a cutoff ≤25 SNPs. All 66 clusters identified by the manual pipeline were identified by the automated system, which found additional clusters with relatedness at the 25 SNP border defined for the study.

The most common reason for additional SNPs called by the automated tool was short mapping segments in otherwise unmapped regions which the manual pipeline excluded from variant calling. The most common reason for additional SNPs called by the manual pipeline was misalignment around indels. As a result, there are isolates that will be called as related by one tool and unrelated by the other. However, this largely affects designation of related or unrelated when the isolate pair is close to the cutoff, and which may have high sensitivity for clustering patients as potentially associated but low specificity for having a true epidemiological link. The cutoff used was designed to be inclusive rather than exclusive, and thus is likely to include epidemiologically unrelated isolates close to the cutoff.

Genome relatedness data identified 66 patient clusters (putative outbreaks) by both methods, the majority of which had not been confirmed through existing IPC methods alone. This corroborates previous findings that genome data provides higher resolution for outbreak detection than existing methods. If this tool was in clinical use, it would rule out the need for investigation of MRSA patients who were clustered by chance in time and space and would provide a priority flag for clusters that required further IPC investigation. The data produced in this study was accessed on several occasions by the IPC team, which resulted in implementation of targeted measures both to reduce spread and to rapidly step-down unrequired actions in cases where suspected outbreaks could be refuted. It was not the aim of the study to implement the automated tool in clinical practice or systematically record the impact of the data provided. This is the focus of a follow-on study that will span an entire year and will track how findings would alter IPC practice and provide an analysis of the consequent economic benefit to the NHS Trust.

The automated nature of the analysis system provides greater standardization and removes interoperator variability compared with research pipelines. This is much needed if genome sequencing it to be used in clinical practice based on the findings of a 13-center proficiency testing evaluation in the Netherlands (8). Centers received two data sets containing 40 genomes each of Klebsiella pneumoniae and vancomycin-resistant Enterococcus faecium, which were analyzed for antimicrobial resistance, sequence type, and outbreak clusters. The reported outbreak clusters revealed discrepancies between centers even when almost identical bioinformatic workflows were used, and some centers failed to detect extended-spectrum beta-lactamase genes and MLST loci. Furthermore, applying a standardized method to determine outbreak clusters on the reported *de novo* assemblies did not result in uniformity of outbreak-cluster composition (8).

There are limitations to the automated tool that need to be overcome before it could support rapid infection control decision-making. The tool had fewer quality control metrics compared to those described previously using the manual research-led method (10). The version evaluated was a standalone genomics tool that was not interfaced with health care providers. As a proof of concept, the tool has since been updated to be capable of outputting to a web-interface on an NHS server through an AIMES environment. This study focused on bioinformatics performance and required additional epidemiological analysis to place patients in time and space. A newer version of the automated tool has functionality supporting epidemiological data upload and integration with the genetic relatedness results to visualize transmission timeline and time-and-place overlap. In future, patient admission and discharge records could be uploaded to a web interface and integrated to present transmission networks with an interactive transmission timeline.

This early study has shown that automated analysis and rapid data generation can inform outbreak detection and exclusion. The automated system generated results without the need for an operator with any bioinformatic training. Its output included reports designed to be consumed and utilized for further action, such as review of time and place overlaps, by Infection Control teams. Our findings provide support for such tools in IPC practice and begin to address one of the barriers to the implementation of MRSA sequencing to enhance the control of infection transmission.

## MATERIALS AND METHODS

### Ethical approval, study setting, patients, and sample identification.

The study had ethical approval from the National Research Ethics Service (ref: 11/EE/0499) and the Cambridge University Hospitals NHS Foundation Trust Research and Development Department (ref: A092428). The study was conducted at the Clinical Microbiology and Public Health Laboratory at the Cambridge University Hospitals NHS Foundation Trust (CUH) in the United Kingdom (UK). Consecutive patients with MRSA-positive samples submitted by three hospitals and 65 GP surgeries during a 9-month period between 24 January and 1 November 2018 were identified using the hospital IT system (EPIC EMR [Hyperspace 2014; Epic Systems Corporation]). Laboratory data were collected on date and place of sampling, and sample type (screen or clinical sample). During the first 3 months, MRSA isolates were retrieved from the freezer archive of the routine laboratory. Isolates were plated onto Columbia Blood Agar (CBA) using a 1 μL loop and incubated overnight at 37°C in air. A single colony was selected for sequencing, and a 10 μL loopful was stored at −80°C in Microbank vials (Pro-lab Diagnostics). During the remaining 6 months, putative or confirmed MRSA-positive culture plates were prospectively retrieved from the laboratory and confirmed as S. aureus using the Staph Latex kit (Pro-lab Diagnostics). A single 2 to 3 mm colony was picked from clinical culture plates using a 1 μL loop. Where colonies were smaller than 2 mm, several colonies were picked. Where bacterial growth was confluent, a 1 μL loopful was taken. If there were several positive plates for one clinical sample, the plate with the least visible background contamination was selected. Isolates were either extracted directly from the clinical plate or subcultured overnight on CBA before processing further. A 10 μL loopful was stored at −80°C in Microbank vials. The first available MRSA isolate from each patient was selected for sequencing, together with all isolates associated with bloodstream infection if this occurred. After de-duplication we identified 789 MRSA isolates cultured from 784 patients.

### Whole-genome sequencing.

Isolates were renumbered with an anonymous study code. DNA was extracted using the Qiagen DNA mini extraction kit and library preparation was performed using the Illumina Nextera DNA flex kit, as described previously (11). Libraries were sequenced on an Illumina MiniSeq with a run time of 13 h using the high output 150 cycle MiniSeq cartridge and the Generate Fastq workflow. Each run contained three controls (no template, positive control (MRSA MPROS0386) and negative control (E. coli NCTC12241)). The study sequences are available in the European Nucleotide Archive (https://www.ebi.ac.uk/ena) and accession numbers are provided in Table S1.

### Quality control metrics.

For the researcher-led manual bioinformatics pipeline, controls and clinical MRSA isolate sequences were required to pass quality control (QC) metrics prior to further analysis, as previously described (10, 11). For the automated tool, isolates were flagged as passed or failed based on having 20× coverage depth over at least 80% of the mapping reference genome and no metrics were used to assess controls.

### Sequence data analysis using manual bioinformatics pipeline.

Bacterial species were defined using Kraken version 1 (https://ccb.jhu.edu/software/kraken/) and the miniKraken database (https://ccb.jhu.edu/software/kraken/dl/minikraken_20171019_8GB.tgz). Multilocus sequence types (ST) were identified for MRSA using ARIBA version 2.12.1, as described at https://github.com/sanger-pathogens/ariba/wiki/MLST-calling-with-ARIBA. MRSA were screened for the presence of *mecA* (accession number HE681097, position 2790560–2792566), *mecB* (accession number AP009486, position 25508:27532) or *mecC* (accession number FR821779, position 35681:37678) using ARIBA, with a minimum percentage identity of 70% required based on Ito et al. 2012 (12), a minimum of 90% of the gene length covered and a depth of coverage no less than 2 standard deviations below the average coverage across the genome (10).

Isolates were mapped to CC22 HO5096 0412 (accession number HE681097). Mapping was performed using SMALT (https://www.sanger.ac.uk/science/tools/smalt-0) with mapping and base calling performed as described in Klemm et al. 2018 (13) with the following modifications: kmer size 13, step size 6. Mobile genetic elements were removed from the alignment using the file available at https://figshare.com/articles/Mobile_genetic_elements_on_the_ST22_strain_HO_5096_0412/7059365 and the script available at https://github.com/sanger-pathogens/remove_blocks_from_aln. Single nucleotide polymorphisms (SNPs) were identified using the script available at https://github.com/sanger-pathogens/snp-sites. The depth and percentage coverage of the mapping reference were determined using the script available at https://github.com/sanger-pathogens/vr-codebase/blob/master/lib/VertRes/Pipelines/Mapping.pm. The number of heterozygous sites >50bp apart was determined using the script available at Github (https://github.com/kumarnaren/mecA-HetSites-calculator). This script also provided depth of coverage and standard deviation of coverage for the *mec* gene, which could be used to calculate whether the *mec* gene had sufficient depth to pass QC (see above).

CC-specific mapping was performed as described above with the following references: CC1 MW2 (accession number BA000033), CC5 N315 (accession number BA000018), CC8 USA300 (accession number CP000255), CC30 MRSA252 (accession number BX571856), CC45 CA347 (accession number CP006044), and CC59 M013 (accession number CP003166). Mobile genetic element files used for these references are available at http://figshare.com/authors/Francesc_Coll/5727779.

### Sequence data analysis using an automated system.

The fully automated tool used here has been updated since our previous report on 17 genomes (6). The cloud-based bioinformatics system version ‘0.1 beta’ self-activated on completion of the 13-h Miniseq run and uploaded raw reads automatically through an on-site computer. This pipeline processed 24 fastq file-pairs generated in a single run and determined relatedness by mapping to a reference MRSA genome (using the same reference as the manual pipeline) where mobile genetic elements were removed as described above. The raw reads were trimmed with Trimmomatic v.0.36-4 to remove low quality bases with a quality score <10 from the ends of each read and filter out reads with <20 average base pair quality. The resulting pool of reads were mapped to a reference with SMALT 0.7.6. Variants were called with Samtools 1.3.1 and Bcftool 1.3.1 with mobile genetic elements masked out. Isolates that passed QC (20× coverage depth over at least 80% of the mapping reference genome) were analyzed for genetic relatedness. Each site of every pair of QC-passed isolates was compared, taking into account evidence supporting both reference and alternative alleles with a proprietary pipeline module. Once the data were uploaded, the automated pipeline took on average 30 min (average taken from three separate runs with a back-database of 52, 53, and 54 sequence runs where in each case relatedness at the SNP level was computed for over 1,000 strains, which took 28 min (m) 28 s (s), 30m9s, and 31m10s, respectively). The resulting data consisted of a summary file and a matrix file in tab-delimited text format.
